# Agility: What It Is, How to Measure It, and How to Use It

**DOI:** 10.1007/s40617-020-00465-4

**Published:** 2020-08-03

**Authors:** Staheli Meyer, Donny Newsome, Timothy Fuller, Kendra Newsome, Patrick M. Ghezzi

**Affiliations:** 1 BCBA-D, University of Nevada, Reno, 1664 N. Virginia St., Reno, NV 89557 USA; 2 Fit Learning™, Reno, NV USA

**Keywords:** Agility, Celeration, Fluency, Precision teaching, Standard celeration chart

## Abstract

A positive and expected by-product of a well-programmed instructional sequence is an escalation of learning, where skills are acquired more quickly as teaching goes on. Despite the importance of this effect in behavior analysis and education, techniques for detecting and analyzing it are rarely observed in practice settings. A behavioral approach to this phenomenon is rooted in the term *agility*, which has persisted in the precision-teaching community as a description of desirable acquisition patterns. Precision teachers have long carried forward a loose definition of agility as “celerating celerations.” Although this definition might succeed in generally orienting practitioners toward the goal of helping people acquire new skills more quickly, its lack of technical specificity has hindered efforts to fully integrate such analyses into practice. In this article, the authors define agility and distinguish it from other concepts common to education and behavior analysis. Further, a tutorial for quantifying and analyzing agility using frequency, celeration, and bounce multipliers is presented in detail. Finally, the practical implications afforded by analyses of agility are delineated.

The “shame of American education” continues (National Assessment of Educational Progress, 2019; Program for International Student Assessment, 2018; Skinner, 1984). Common Core State Standards (CCSS) provide a road map to college readiness by identifying the minimum competency level expected for each grade in school (i.e., grades K through 12; Haager & Vaughn, 2013). Ideally, students are supported in meeting these increasingly rigorous standards in each grade and complete high school with all the valuable skills, habits, and knowledge needed to attend college and participate in the modern workforce. In practice, however, too many students find themselves failing in the education system despite the intentions of the CCSS road map. For example, the developers of the American College Test (ACT) college readiness exam (Dougherty & Fleming, 2012) report that “the majority of students who finish high school do not graduate college and career ready” (p. 1) and that low-income students are at an even higher risk, with only 27% meeting benchmarks in reading, 16% in mathematics, and 11% in science.

One of the main problems the ACT research group points to is the fact that students who fall behind tend to stay behind. Dougherty and Fleming (2012) found that among students who were “off track” in 8th grade (35%–41% of all students), only 19% met 12th-grade benchmarks in mathematics, 29% in reading, and 32% in science. For 8th graders deemed “far off track” (12%–42% of all students), only 3% meet 12th-grade benchmarks in mathematics, 10% in reading, and 6% in science. To describe the challenges of getting students back on the CCSS road map once they have taken a wrong turn, the following reasoning was offered:

Closing students’ preparation gaps relative to college and career readiness requires students who are academically behind to grow *faster* than students who are ahead of them. The lagging students must do double duty, catching up on content that they missed earlier while mastering newly taught curriculum. Students who are already on track do not carry this extra burden. (Dougherty & Fleming, 2012, pp. 2–3)

Implicit in the reasons given for why students who fall behind stay behind is a self-evident solution. Simply put, we need a way to help students learn more quickly. Those involved in teaching and learning have a keen interest in creating a learning context in which learners come to acquire new skills more quickly. Practitioners strive not only for uniform gains across time but also for multiplicative gain. That is, they serve as agents in the transformation of learners from fledglings to masters of acquisition—from linear to exponential learning. Efficient behavior change occurs multiplicatively, not just additively (Lindsley, 1997). As such, acquisition data indicating a linear relationship between time spent in instruction and skills mastered would be viewed as a missed opportunity.

The solution to getting students on track is to create a learning environment where skills multiply over time. A linear learning trajectory is not enough when the objective is rapid remediation. Despite the importance of promoting a more efficient acquisition of skills in applied behavior analysis and education, techniques for detecting and analyzing efficiency are rarely observed. Developing solutions to help students catch up will require a method for quantifying how quickly a student is learning and a conceptual framework to inform techniques for accelerating the speed at which a student learns. A thorough understanding of the concept of agility, how to measure it, and how to use it may prove valuable in the service of that need. The following tutorial on behavioral agility attempts to give readers a new road map toward more efficient educational practices.

## Defining Agility

The term *agility* has persisted as a description of increasingly efficient skill acquisition, an indisputably desired outcome of effective instruction (Lindsley, 2000). Much like other behavior-analytic terms (e.g., reinforcement, celeration, and momentum), the precision-teaching community has borrowed this terminology from other domains. In borrowing such terms, the concepts are used metaphorically, incorporated into the lexicon, and given a precise and technical meaning. The present article attempts to define and distinguish agility from other concepts to clarify and bring precision to its usage. In doing so, the term moves beyond a description of physical action and is used metaphorically to describe characteristics of learning. Such a description is anchored to metrics for quantifying changes in acquisition. The authors present a tutorial for quantifying agility using frequency, celeration, and bounce multipliers, as well as the practical implications afforded by analyses of agility that follow.

Lindsley (2000) suggests, “Once agile (steep celeration), a learner feels ready for any learning challenge” (p. 107). He further suggested that agility could be thought of as “fast, smooth, accurate, automatic, skilled performance” (p. 107). In moving from metaphor to technical usage, Lindsley suggested that agility could be anchored to the measure of *celerating celerations.* This is depicted on the standard celeration chart (SCC) as increasingly steeper celerations, or learning slopes, across acquisition targets.

Lin and Kubina (2015) show data indicative of agile acquisition. The researchers taught a young girl with autism spectrum disorder motor imitation using timed practice. The acquisition of these imitative responses shows celerations becoming successively steeper. According to Lin and Kubina, as the learner “became fluent with past sets of behaviors, she learned the new sets more quickly than the previous ones” (p. 13). They describe the phenomenon of quicker learning as agility. Agility has also been measured in other ways. Neely (2003) proposed that agile learning could be characterized by quickly reaching goals and requiring fewer practice opportunities to reach them.

**Fig. 1.**
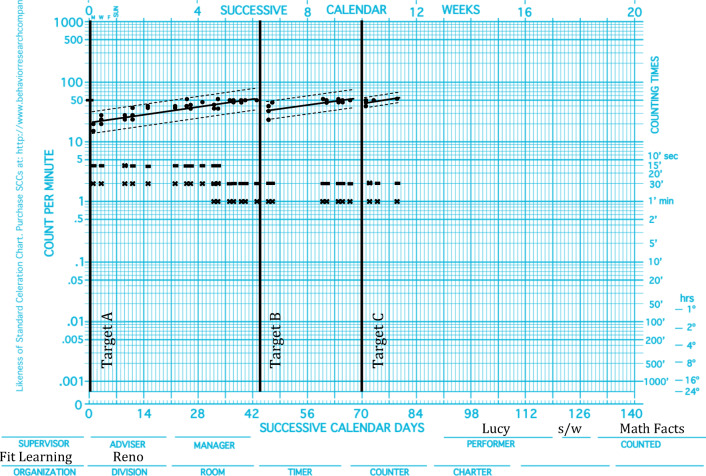
Lucy’s math facts acquisition data

Figure 1 depicts a sample SCC of acquisition data that would be described as agile. Lucy received services for math remediation. Targets A, B, and C were composed of an equal number of facts, with no facts overlapping between the target sets. There are visually distinguishable acquisition patterns across Target A and Targets B and C. Target A was the first set of math facts Lucy acquired in her training sequence; after achieving performance standards on Target A, Targets B and C reached performance standards in less time and with fewer practice opportunities.

Precision teachers have come to consistently describe faster, more efficient acquisition as agility and view the concept as important and useful to achieve practical goals. In this context, *celeration of celeration* is a reasonable starting point, as it generally orients practitioners toward the goal of helping people acquire new skills more quickly. However, its lack of technical specificity has hindered efforts to fully integrate such analyses into practice. A concept this important to explaining the goals and outcomes of precision teaching is worthy of a more detailed definition. What follows is an attempt to provide a more precise, detailed, and expansive definition of agility.

## Distinguishing Agility From Fluency

A detailed definition of agility will be built upon the analogical assentation that “fluency is to frequency” as “agility is to celeration” (Johnson & Street, 2013; Lindsley, 2000). A precise and consistent conceptualization of agility cannot be achieved without distinguishing agility from what it is not. Care must be taken to avoid redundancy with the fluency construct. Distinguishing agility from fluency is a difficult task to accomplish, as the fluency concept has considerable scope and generally deals with similar events of interest. Additionally, the metaphorical ways in which agility has been discussed leave much room for interpretation and are often difficult to parse from the ways in which fluency is described.

The general basis for the distinction is that, whereas fluency is applied to the acquisition of a particular target, the concept of agility captures the relationship across targets. Further, agility is reserved as a comparison between related skills, and application is reserved as a relationship between a component skill and a composite skill. In this context, component skills refer to those constituent actions that participate in tasks that require cumulative participation to accomplish.

Fluency is a term commonly used to describe specific features of a class of behavior (Johnson & Layng, 1996). Binder (1996) describes fluency as an outcome in which, when a learner performs a task accurately and at a certain frequency, several affordances are observed. For instance, Binder (1996) points to the observation that learners persist or endure in the task, often in what would appear to be distracting environing circumstances, and can apply these skills to new situations. Fluency, therefore, is related to the optimal frequency of accurate responding, which provides a quantifiable, objective, verifiable measure of the concept. Further, the functional outcomes said to indicate the fluency of a response class are also measurable. Kubina and Yurich (2012) acknowledge that up to the date of publication of their precision-teaching text, the research in the area of fluency outcomes has been primarily focused on the following metrics: “task maintenance, endurance, stability, application, and generativity” (p. 334).

The concept of fluency has tremendous utility for describing the relationship between a measure of learning (i.e., celeration) and important functional outcomes (i.e., retention, endurance, and stability; Binder, 1996; Haughton, 1972; Johnson et al., 2004). An orientation toward fluency changes practitioners’ behavior in advantageous ways, usually toward more sensitive and predictive behavioral measures and charting conventions. Furthermore, practitioners are better situated to address the concerns of their clients and speak to the amelioration of these concerns through the measures employed.

To reiterate, our position is that fluency is applied with respect to the measurement and acquisition of a particular target, whereas the concept of agility captures practical interest in the relationship across similar, directly trained targets. That is, agility can be used to describe the change in acquisition from Target A to acquisition on subsequent Targets B, C, D, E, and so on. In this framework, it can be said that agility, although based on all of the same basic performance measures as fluency, provides a comparative analysis across similar, directly trained targets. Agility, as described previously, allows for observations and measures of the effect that achieving fluency on one target leads to improved acquisition on similar, subsequent targets. For example, the learner who has mastered the first five letters of the alphabet may show a measurably different acquisition on the next five letters of the alphabet.

*Application* is a term used to describe the relationship between component and composite skills (Johnson & Layng, 1996). In practice, this term is used to describe performance on Target A, as well as performance on some composite skill, often untrained, of which Target A is a component. For example, writing digits would be considered a component skill necessary to solve a long-division problem (i.e., the composite skill). An agility analysis is not considered part of the relationship between writing your digits and solving multidigit division problems, as this relationship is more akin to application. (See Johnson and Layng, 1996, pp. 180–181, for a historical discussion of component-composite relations and their relationship to application.) Agility describes the relationship between acquisition on Target A and acquisition on subsequent targets—Targets B, C, D, E, and so on. Reserving the term *application* for describing relationships between component skills and an untrained composite skill and the term *agility* for describing relationships between acquisition on similar, directly trained targets retains these as conceptual distinguishable and precise.

With these distinctions, it can be said that agility, although based on similar performance measures as fluency, provides a different analysis—one of comparison across targets. Thus, the extended analogy is fluency is to frequency, celeration, and bounce within a target, as agility is to change in frequency, celeration, or bounce across targets.

## Quantifying Agility

Agility, when viewed this way, is also amenable to quantification. Each of the measures commonly applied to the quantification of fluency (e.g., frequency, celeration, bounce) can be used in the calculation of agility by placing it in a ratio with the same measures taken on all previous or subsequent targets (see Table 1). The proposed metric for quantification comes from conventions used in comparative analyses. They are described in detail (see Datchuk & Kubina, 2011; Pennypacker, Gutierrez, & Lindsley, 2003) as “frequency multipliers” and “celeration multipliers,” also respectively called “jumps” and “turns” (see Graf & Lindsley, 2002; Kubina & Yurich, 2012). In each case, acquisition can be compared by using a base formula to calculate the ratio of frequencies, celerations, and bounce changes across targets (see Eq. 1).

1 $$ \mathrm{Measure}\ \mathrm{of}\ \mathrm{agility}=\frac{\mathrm{Larger}\ \mathrm{measure}\ \mathrm{of}\ \mathrm{fluency}}{\mathrm{Smaller}\ \mathrm{measure}\ \mathrm{of}\ \mathrm{fluency}} $$

**Table 1:** Measures Applied to the Quantification of Fluency and Agility

Measure of Fluency	Measure of Agility
Frequency	Frequency multiplier
Celeration	Celeration multiplier
Bounce	Bounce multiplier

Any measure of acquisition on a given target may be used this way to quantify one aspect of agility. To return again to the analogy, regarding the quantification of agility, fluency is to frequency, celeration, bounce, and so on, as agility is to the frequency multiplier, celeration multiplier, bounce multiplier, and so on.

**Fig. 2.**
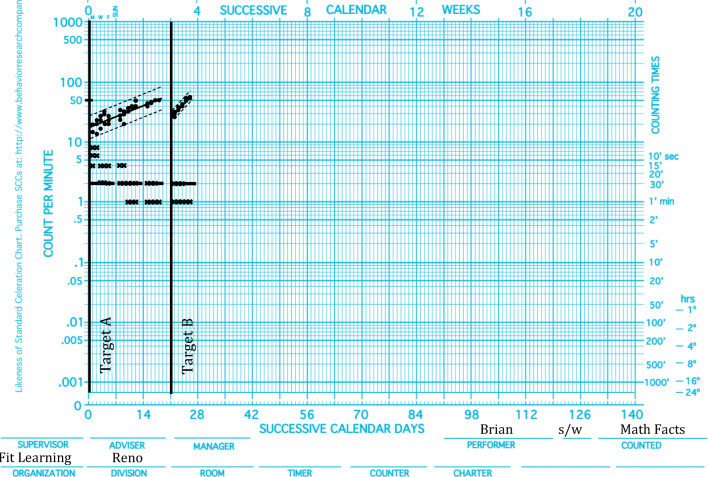
Brian’s math facts acquisition data

Figure 2 uses a hypothetical data set to demonstrate agility in the acquisition of math facts for Brian. Targets A and B had an equal number of facts, with no facts overlapping between the two sets. Acquisition on Target A was measured as a celeration of ×1.5, a base frequency of 16, a bottom frequency of 14, a top frequency of 50, and a bounce of ×1.8. Acquisition on Target B was measured as a celeration of ×3.0, a base frequency of 28, a bottom frequency of 28, a top frequency of 56, and a bounce of ×1.2 (see Table 2 for a side-by-side look at these measures). Visual inspection of these data provides a number of indicators of agile learning. Simply put, Target B was mastered more quickly than Target A. To substantiate this assertion, we can set about quantifying agility as described previously. To do so, one creates a ratio by dividing the larger number by the smaller number and indicating the sign of change: × for multiplying change or ÷ for dividing change (see Table 3 for these calculations).

**Table 2:** Side-by-Side Comparison of Brian’s Math Facts Acquisition

Measure	Target A	Target B
Celeration	×1.5	×3.0
Base frequency	16	28
Bottom frequency	14	28
Top frequency	50	56
Bounce	×1.8	×1.2

**Table 3:** Multiplier Calculations of Brian’s Math Facts Acquisition

Measure	Multiplier Formula Target A to Target B	Multiplier Calculation Target A to Target B	Multiplier Value Target A to Target B
Celeration	Divide the larger celeration by the smaller celeration and show the sign of change.	(×3.0) ÷ (×1.5)	×2.0
Base frequency	Divide the larger base frequency by the smaller base frequency and show the sign of change.	(28) ÷ (16)	×1.75
Bottom frequency	Divide the larger bottom frequency by the smaller bottom frequency and show the sign of change.	(28) ÷ (14)	×2.0
Top frequency	Divide the larger top frequency by the smaller top frequency and show the sign of change.	(56) ÷ (50)	×1.12
Bounce	Divide the larger bounce by the smaller bounce and show the sign of change.	(×1.8) ÷ (×1.2)	÷1.5

### Celeration Multiplier

The celeration for Target A is measured at ×1.5; the celeration for Target B is measured at ×3.0. The larger celeration (×3.0) is divided by the smaller celeration (×1.5). Target B was the larger measure, meaning the change was multiplicative.

$$ \mathrm{Celeration}\ \mathrm{multiplier}=\frac{\mathrm{Larger}\ \mathrm{celeration}}{\mathrm{Smaller}\ \mathrm{celeration}}\kern0.3em \times 2.0=\frac{\times 3.0}{\times 1.5} $$

### Base Frequency Multiplier

The base frequency for Target A is measured at 16; the base frequency for Target B is measured at 28. The larger base frequency (28) is divided by the smaller base frequency (16). Target B was the larger measure, meaning the change was multiplicative.

$$ \mathrm{Base}\ \mathrm{frequency}\ \mathrm{multiplier}=\frac{\mathrm{Larger}\ \mathrm{base}\ \mathrm{frequency}}{\mathrm{Smaller}\ \mathrm{base}\ \mathrm{frequency}}\kern0.3em \times 1.75=\frac{28}{16} $$

### Bottom Frequency Multiplier

The bottom frequency for Target A is measured at 14; the bottom frequency for Target B is measured at 28. The larger bottom frequency (28) is divided by the smaller bottom frequency (14). Target B was the larger measure, meaning the change was multiplicative.

$$ \mathrm{Bottom}\ \mathrm{frequency}\ \mathrm{multiplier}=\frac{\mathrm{Larger}\ \mathrm{bottom}\ \mathrm{frequency}}{\mathrm{Smaller}\ \mathrm{bottom}\ \mathrm{frequency}}\kern0.3em \times 2.0=\frac{28}{14} $$

### Top Frequency Multiplier

The top frequency for Target A is measured at 50; the top frequency for Target B is measured at 56. The larger top frequency (56) is divided by the smaller top frequency (50). Target B was the larger measure, meaning the change was multiplicative.

$$ \mathrm{Top}\ \mathrm{frequency}\ \mathrm{multiplier}=\frac{\mathrm{Larger}\ \mathrm{top}\ \mathrm{frequency}}{\mathrm{Smaller}\ \mathrm{top}\ \mathrm{frequency}}\kern0.3em \times 1.12=\frac{56}{50} $$

### Bounce Multiplier

The bounce for Target A is measured at ×1.8; the bounce for Target B is measured at ×1.2. The larger bounce (×1.8) is divided by the smaller bounce (×1.2). Target A was the larger measure, meaning the sign of change is division.

$$ \mathrm{Bounce}\ \mathrm{multiplier}=\frac{\mathrm{Larger}\ \mathrm{bounce}}{\mathrm{Smaller}\ \mathrm{bounce}}\kern0.5em \div 1.5=\frac{\times 1.8}{\times 1.2} $$

The result of each ratio will be either a frequency, celeration, or bounce multiplier. This multiplier method produces values of greater than or equal to 1, wherein a multiplier of 1 indicates no change, and a higher score indicates a greater degree of change. A multiplication symbol indicates a change up the logarithmic scale (i.e., acceleration or more bounce), and a division symbol indicates change down the logarithmic scale (i.e., deceleration or less bounce). Improvement is considered a change greater than 1.0 for the celeration, bounce, and frequency multipliers. The degree of improvement and the significance of change across targets (i.e., agility) can be evaluated in the same way change is evaluated within targets (i.e., celeration). Kubina and Yurich (2012) suggest the following classifications: Change of ×1.0–×1.25 is unacceptable, change of ×1.25–×1.4 is acceptable, change of ×1.4–×1.8 is robust, change of ×1.8–×2.0 is e×ceptional, change of ×2.0–×3.0 is massive, and change of ×3.0+ is supermassive (see Kubina & Yurich, 2012, Chapter 6, for classifications of the magnitude of behavior change). The same values and corresponding classifications can also be used to measure the magnitude of improvement across similar, directly trained targets (Table 4).

**Table 4:** Magnitude of Change Classifications Based on Multiplier and Divider Values

Multiplier Value Range	Divider Value Range	Percentage Change	Change Classification
×3.0+	÷3.0+	201%+	Supermassive
×2.0–×3.0	÷2.0–÷3.0	101%–200%	Massive
×1.8–×2.0	÷1.8–÷2.0	80%–100%	Exceptional
×1.4–×1.8	÷1.4–÷1.8	40%–79%	Robust
×1.25–×1.4	÷1.25–÷1.4	25%–40%	Acceptable
×1.0–×1.25	÷1.0–÷1.25	0%–24%	Unacceptable

Quantification from Fig. 2 demonstrates agility on all multiplier measures. Agile learning can be observed on some but not all dimensions, however. To return to Lucy’s acquisition (Fig. 1), the practicality of having multiple agility indicators at the practitioner’s disposal is illustrated. See Table 5 for the calculation comparisons for Targets A, B, and C. Results indicate improvements across targets for base frequencies, bottom frequencies, and bounce but not for the celeration and top frequency multipliers across targets (see Tables 6 and 7 for side-by-side comparisons and multiplier calculations and classifications).

**Table 5:** Multiplier Calculations of Lucy’s Math Facts Acquisition

Measure	Multiplier Formula	Multiplier Calculation Target A to Target B	Multiplier Value Target A to Target B	Multiplier Calculation Target B to Target C	Multiplier Value Target B to Target C
Celeration	Divide the larger celeration by the smaller celeration and show the sign of change.	(×1.2) ÷ (×1.2)	×1.0	(×1.2) ÷ (×1.2)	×1.0
Base frequency	Divide the larger base frequency by the smaller base frequency and show the sign of change.	(24) ÷ (16)	×1.5	(52) ÷ (24)	×2.2
Bottom frequency	Divide the larger bottom frequency by the smaller bottom frequency and show the sign of change.	(24) ÷ (16)	×1.5	(40) ÷ (24)	×1.57
Top frequency	Divide the larger top frequency by the smaller top frequency and show the sign of change.	(52) ÷ (52)	×1.0	(52) ÷ (52)	×1.0
Bounce	Divide the larger bounce by the smaller bounce and show the sign of change.	(×1.7) ÷ (×1.5)	÷01.13	(×1.5) ÷ (×1.3)	÷1.15

**Table 6:** Side-by-Side Comparison of Lucy’s Math Facts Acquisition

Measure	Target A	Target B	Target C
Celeration	×1.2	×1.2	×1.2
Base frequency	16	24	52
Bottom frequency	16	24	40
Top frequency	52	52	52
Bounce	×1.7	×1.5	×1.3

**Table 7:** Multiplier Calculations of Lucy’s Math Facts Acquisition

Measure	Multiplier Calculation	Multiplier Value	Change Classification
Target A to Target B			
Celeration	(×1.2) ÷ (×1.2)	×1.0	Unacceptable
Base frequency	(24) ÷ (16)	×1.5	Robust
Bottom frequency	(24) ÷ (16)	×1.5	Robust
Top frequency	(52) ÷ (52)	×1.0	Unacceptable
Bounce	(×1.7) ÷ (×1.5)	÷1.13	Unacceptable
Target B to Target C			
Celeration	(×1.2) ÷ (×1.2)	×1.0	Unacceptable
Base frequency	(52) ÷ (24)	×2.2	Massive
Bottom frequency	(40) ÷ (24)	×1.7	Robust
Top frequency	(52) ÷ (52)	×1.0	Unacceptable
Bounce	(×1.5) ÷ (×1.3)	÷1.15	Unacceptable

Upon visual inspection, Lucy’s acquisition appears agile. However, the traditional metric of agility as *celeration of celeration* would exclude this example within the limits of the original definition. By extending the quantification of agility to include all possible multiplier values across all dimensions of fluency, rather than just celeration multipliers, the scope is broadened to include a greater variety of acquisition patterns within a pragmatic notion of agility. Not all measures of agility must necessarily be affirmed to say agility has been observed, as it is not always the clinical goal to change all dimensions. Clinical goals guide the selection of agility quantification metrics. When the desired clinical goal is a change in top frequencies, bottom frequencies, or base frequencies, a frequency multiplier would be the calculation of choice. If the clinical goal is to improve the bounce (i.e., reduce variability), a bounce multiplier is the most informative calculation. Additionally, the celeration multiplier calculation captures the classical description of agility proposed by Lindsley (2000)—that is, celeration of celeration.

Although the example of Lucy’s performance is plotted on a daily per-minute chart, the same multiplier calculations can be applied to the family of charts. Such scalability across both the family of charts and the equation permit evaluations of agile performance to occur across any standard unit of time, from minute by minute, to year by year, to decade by decade, and so on.

## Implications for Practice

Quantifying agility affords scientist-educators a way by which teaching and learning can be measured, evaluated, and improved. The implications that quantifying agility has on teaching and learning are vast and include ways of selecting agile learning (i.e., reinforcing agile performance), programming for agility, and evaluating curricula for the promotion of agility.

The aforementioned methods for calculating agility have used a post hoc analysis using mathematical equations for quantification. Using these formulas will yield the most precise analysis of agility and is therefore recommended for research purposes. Those evaluating agility in practice settings, however, may do so without interrupting their programming to make calculations. The SCC supports real-time decision making on the basis of visual inspection when performance measures are charted in real time. In the same way educators can reinforce responding that celerates without calculating celeration lines, so too can educators detect and react to patterns of agility without formal calculations. When suggesting how we can estimate celeration lines, Pennypacker et al. (2003), the authors of the *Handbook of the Standard Celeration Chart*, state,

We can draw such lines with surprising accuracy . . . the line we have drawn is an approximation to the “line of best fit” which requires complex mathematical operations best performed on a computer. Experience has shown that with little practice, we can draw freehand celeration lines that are virtually indistinguishable from those drawn with the aid of a computer. (p. 52)

**Fig. 3.**
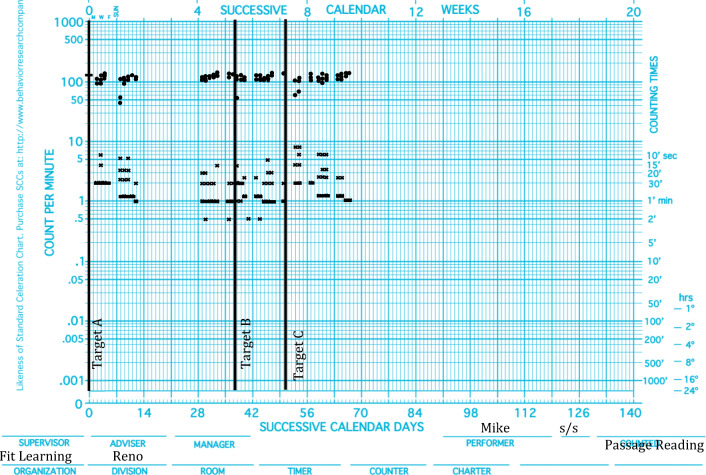
Mike’s passage-reading acquisition data

**Fig. 4.**
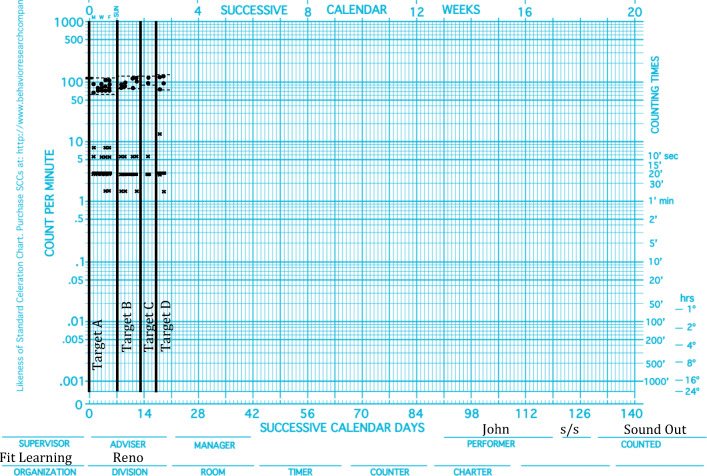
John’s sounding-out-words acquisition data

In the same way that educators can visually inspect patterns of performance and estimate celeration, so too can they estimate patterns of agility. Equipped with the concept and metrics of agility, educators can evaluate and promote agile acquisition. Immediately following timed practice, performance can be charted, evaluated, and consequated. Reinforcers can be delivered contingent on changes in celeration, top frequencies, bottom frequencies, base frequencies, or bounce. For example, a scientist-educator reviewing Mike’s acquisition data (see Fig. 3) may decide that the magnitude of change across targets is unacceptable and may program reinforcement for increasing changes over time on the clinically relevant dimension. In Mike’s case, a scientist-educator may choose to set the criterion for reinforcement as increases in base frequencies. An educator reviewing John’s data (see Fig. 4) may conclude that the change in bounce across targets is unacceptable and program reinforcement for more stable responding across performances.

Detecting agile patterns may also inform curriculum adjustments. If learning is occurring more quickly, targets can be increased in their respective complexity. For example, a scientist-educator reviewing Lucy’s acquisition (see Fig. 1) may program for more material in subsequent targets; this change is sometimes called a “curriculum leap” (Kubina & Yurich, 2012). Detecting agile acquisition informs educators regarding a learner’s preparedness for curricula in other instructional environments. Quantifying changes in learning across targets has predictive value, by allowing projections with respect to how long a learner will require to achieve mastery in a given academic domain.

Curricula can be evaluated, in part, on how well they reliably produce agile learning. If curricula do not produce agile learning, their effectiveness and efficiency can be reconsidered. With agility as an explicit goal, curricula can further be constructed in ways that are scalable to accommodate increasingly agile acquisition as students move through the content. The Morningside model of generative instruction (Johnson et al., 2004) math sequence is an example of such scalability. The curriculum can be arranged to teach between 8 and 190 new math facts at a time.

## Summary

When researchers and scientist-educators are attentive to patterns of agility, the adequacy of a learning environment may be assessed not only by the extent to which a single target or lesson is acquired but also on how doing so impacts subsequent learning patterns. A metric is given by which the practical aim of creating learning contexts where learners come to acquire new skills faster can be evaluated and enhanced. The array of multiplier measures is offered as a practical and reliable means of detecting agility in clinics, classrooms, and research settings. Adoption of these measures and familiarity with their visual representations on the SCC are thus encouraged to the extent that their use may improve scientist-educators’ decision making, as well as guide the development of an elaborated behavioral account of learning more quickly as teaching goes along.

When the agility concept is situated at the level of comparison across similar, directly trained targets, quantified by multipliers, it is conceptually and mathematically distinguishable from fluency and application. At the same time, this understanding of agility as a mathematical and conceptual derivative of fluency illuminates the close interrelationship among these concepts: There is no agility without fluency.
